# Palliative care on the radiation oncology ward—improvements in clinical care through interdisciplinary ward rounds

**DOI:** 10.1007/s00066-022-01989-0

**Published:** 2022-08-11

**Authors:** Michael Oertel, Renate Schmidt, David Rene Steike, Hans Theodor Eich, Philipp Lenz

**Affiliations:** 1 Department of Radiation Oncology, University Hospital Muenster, Albert-Schweitzer-Campus 1, 48149 Muenster, Germany; 2 Department of Palliative Care, University Hospital Muenster, Albert-Schweitzer-Campus 1, 48149 Muenster, Germany

**Keywords:** Medical education, Palliation, Interdisciplinary cooperation, Radiotherapy, Radio-oncology

## Abstract

**Introduction:**

Palliative care is essential for patients with terminal diseases and aims at effective symptom control. This may stand in opposition to radiation treatment as an oncological treatment modality. The hereby presented work demonstrates the successful integration of a palliative care service in the radiation oncology ward.

**Methods:**

Since 2015, 1018 patients were seen by the palliative care service on the radiation oncology ward and have been analyzed in this single center study. To assess teaching efficacy of the consultation service, a survey was conducted among 15 radiation oncology residents.

**Results:**

Cooperation between the two departments proved to be efficient with rising patient numbers. Palliative care was able to guide appropriate postdischarge care with the number of patients dying on the radiation oncology ward decreasing significantly (*p* = 0.009). The main topics for consultation were pain medication (92.3%), organization of postdischarge care (92.3%), and psycho-oncological support (84.6%). Most residents had a positive image of the palliative care service and consented on adjectives like “enriching”, “empathic”, “collegial”, “professionally founded”, and a “low threshold for consultation”. All participants agreed that cooperation deepened their knowledge on palliative care.

**Conclusion:**

A synergistic cooperation between a palliative care consultation service and a radiation oncology department addresses patient symptoms on an individual level. It confers advanced knowledge on palliative care which is essential for resident education and patient treatment.

**Supplementary Information:**

The online version of this article (10.1007/s00066-022-01989-0) contains supplementary material, which is available to authorized users.

## Introduction

Radiation treatment (RT) constitutes a cornerstone of cancer therapy with around 50% of oncological patients undergoing radiation during the course of their disease [1, 2]. Despite technical and conceptual innovations in the field [3], palliative concepts remain pivotal and constituted 41% of RT series in an analysis of the surveillance, epidemiology, and end results database (SEER) [4]. The use of RT also extends to the end-of-life period with a systemic review describing a rate of 5–10% among patients dying of cancer in the last 30 days of life [5]. This finding corresponds to another SEER analysis estimating the percentage for RT in the last 30 days of life to be 7.6% in a collective of about 200,000 patients dying of lung, breast, prostate, colorectal, or pancreas cancer, which demands a carefully considered balance of therapeutic interventions and best supportive care concepts [6].

Palliative patients may reveal a complex variety of symptoms, both on a physiological and psychological level, calling for holistic clinical care [7, 8]. Despite this, the abilities of physicians to manage the symptom burden of their patients vary considerably being highest for (somatic) pain but dropping regarding psychological support [9, 10]. In general, palliative care (PC) is of cardinal importance for everyday clinical practice; in a web-based survey of the German Society of Radiation Oncology (DEGRO), 84.4% of respondents answered to be in need of palliative care consultation service often or very often during their daily routine [10]. Previous publications have demonstrated feasibility of cooperation between a palliative care consultation service (PCCS) and a radiation oncology (RO) department [11, 12], yet many questions on the best way for implementation remain unanswered.

The present work intends to analyze the symptom burden of palliative patients on a RO ward in a large collective and thereby derives requirements for effective PC. We describe a multidisciplinary palliative concept based on regular palliative/radio-oncological ward rounds and its integration. Furthermore, educational demands of residents are investigated, and acceptance of the proposed model is assessed.

## Materials and methods

### Study design

The study was designed as a retrospective, single-center study at our institution, where a PCCS was established in May 2015. For the following study, we focused on patient data from January 5, 2015 to August 6, 2021. To analyze the numbers of patients dying on the RO ward, supplementary data were collected comparing the time span before the implementation of PCCS (2010–2015) and the period after the introduction of PCCS (2016–2020). The study was conceptualized by the last author and approved by the local institutional review board (protocol code 2017-636-f-S).

### Patients

Patients with advanced life-limiting and progressive disease were referred to the PCCS by the RO ward physicians when identifying PC needs. Regular weekly ward rounds were undertaken by the PCCS, treating radiation oncologists, and nurses in order to identify patients with need for (specialized) PC. After referral, a PC physician or nurse performed a detailed assessment including symptom burden, psychosocial demands, and spiritual distress. This assessment resulted in a supportive treatment plan carried out by a multiprofessional PC team, involving various professions (physiotherapy, ergotherapy, psychologists, clergy, social service, music therapy), simultaneously to radiation treatment. Consequently, daily interventions could include a wide spectrum like additional ward rounds aimed at symptom relief, consulting of treating physicians concerning medication and supportive care, social services including organization of further (palliative) care after discharge, physiotherapy and ergotherapy sessions, and spiritual as well as psychological counseling. The exact orientation and intensity of the supportive therapy varied depending on the patient’s clinical condition and individual needs. As a consultation service, the PCCS did not interfere directly with decisions on the RO treatment schedule and duration, but could formulate recommendations.

All data were electronically available using the hospital information system Orbis-OpenMed® (Agfa Healthcare, Mortsel, Belgium).

### Radiation therapy

All RO treatments were carried out according to institutional standards adapted to the respective treatment situation on the discretion of the department of RO. There were no exclusion criteria concerning treatment specifications (advanced primary vs. metastatic disease, stereotactic vs. fractionated RT, normo- vs. hypofractionated RT). For treatment series, either a TrueBeam linear accelerator (Varian Medical Systems, Pao Alto, CA, USA) or a Tomotherapy machine (Accuray, Sunnyvale, CA, USA) was used.

### Survey

An 18-item questionnaire was developed by two senior physicians in PC and RO, respectively (first and last author of the present work) and was filled out by residents in RO after their obligatory ward rotation. The questions aimed at palliative knowledge as well as use of and views on the PCCS . The survey was completed anonymously on Lime Survey (Lime Survey, Hamburg, Germany) and answers were analyzed using Excel for Mac (Microsoft Cooperation, Redmond, WA, USA) and SPSS version 28 (IBM, Armonk, NY, USA).

### Statistics

Continuous variables are summarized by the mean values and standard deviations, whereas categorical variables are presented as absolute numbers and relative frequencies. Pain was assessed as a binary variable “pain existent” (0 = nonexistent, 1 = any manifestation from light to unbearable) and graded via a numeric rating scale (0–10) afterwards. Pain assessment was performed at the initiation by the PCCS and 72 h afterwards. Continuous parameters were analyzed by means of a Wilcoxon–Mann–Whitney test which was also used to compare the numbers of dying on the RO ward 2010–2015 with 2016–2020. For categorical variables, a χ^2^ test was employed. Two-sided *P*-values of ≤ 0.05 were considered statistically significant. The statistical analysis of the data was performed using the SPSS Software (IBM SPSS Statistics for Windows, Version 28.0, Armonk, NY, USA) and the SAS Software (Version 9.4, SAS Institute Inc., Cary, NC, USA).

## Results

**Fig. 1:**
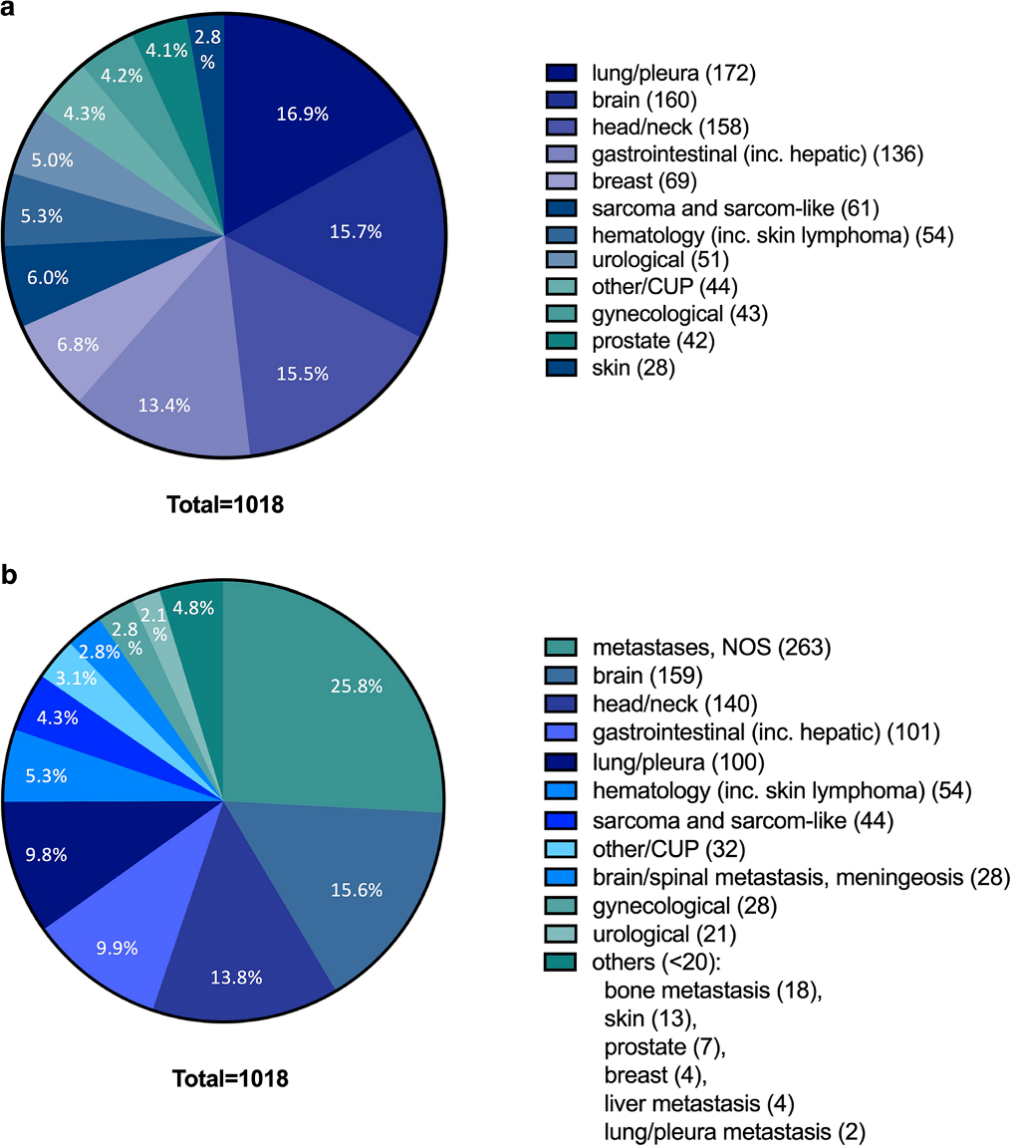
Overview on tumor entities. Relative numbers of treated tumors according to primary (**a**) or treatment situation including metastases (**b**). *CUP* cancer of unknown primary, *NOS* not otherwise specified, *inc.* including

Overall, 1018 patients in the RO department were seen by the PCCS between 2015 and 2021 (Table 1, Fig. 1), 77 (7.6%) of whom were only counseled once on an outpatient basis. The remaining 941 (92.4%) patients were accompanied on the ward.

**Table 1:** Demographic data of the study population

Patients	*n*
*Total*	1018
*Age*	Mean: 65.8 years
Inpatient	941 (92.4%)
Outpatient	77 (7.6%)
Length of stay	Mean: 30 days ± 20 days
Time to integration	Mean: 8 days ± 14 days
Length of cotreatment	Mean: 22 days ± 15 days

**Fig. 2:**
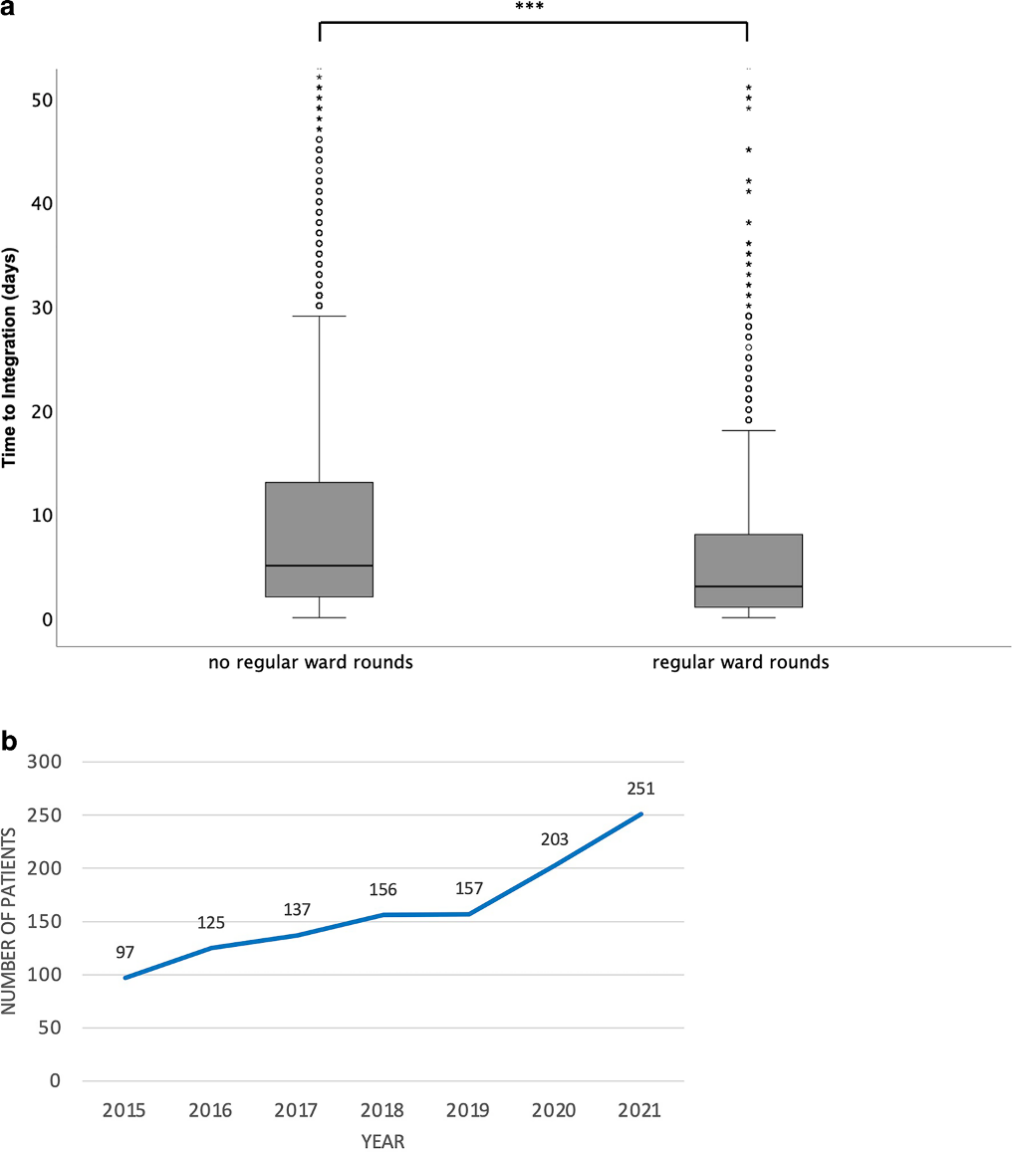
Development of palliative care consultation service (PCCS). **a** The time to integration of PCCS could be decreased significantly by regular ward rounds (mean: 11 ± 20 vs. 8 ± 14 days, *p* < 0.001). **b** Number of patients treated with the aid of PCCS increased continuously from 97 in 2015 to 251 in 2021

Mean age of patients was 65.8 ± 13 years (range 19–95 years) and mean length of stay in the acute care hospital was 30 ± 20 days. The most prevalent primary entities were lung/pleura tumors (16.9%), primary brain tumors (15.7%), and head and neck tumors (15.5%; Fig. 1a). When considering metastases and primary tumors, the most prevalent treatment indications were metastases, not otherwise specified (25.8%), brain tumors (15.6%), and head and neck tumors (13.8%; Fig. 1b). Pain at the time of admission was frequently seen with 20.4% of patients reporting this symptom. Of the 192 patients presenting with pain at time of admission, 177 improved (after 72 h), 8 revealed no changes, and 7 had no follow-up data. Pain intensity decreased from a median value of 6 on the numeric rating scale (range 2–10) to 2 (range 0–8; Supplementary Fig. 1). The mean length of stay under cotreatment by the PCCS was 22 ± 15 days (Table 1). In contrast to patients in other departments than RO (*n* = 3254), the time to integration could be reduced significantly by regular ward rounds (mean: 11 ± 20 vs. 8 ± 14 days, *p* < 0.001; Fig. 2a).

**Fig. 3:**
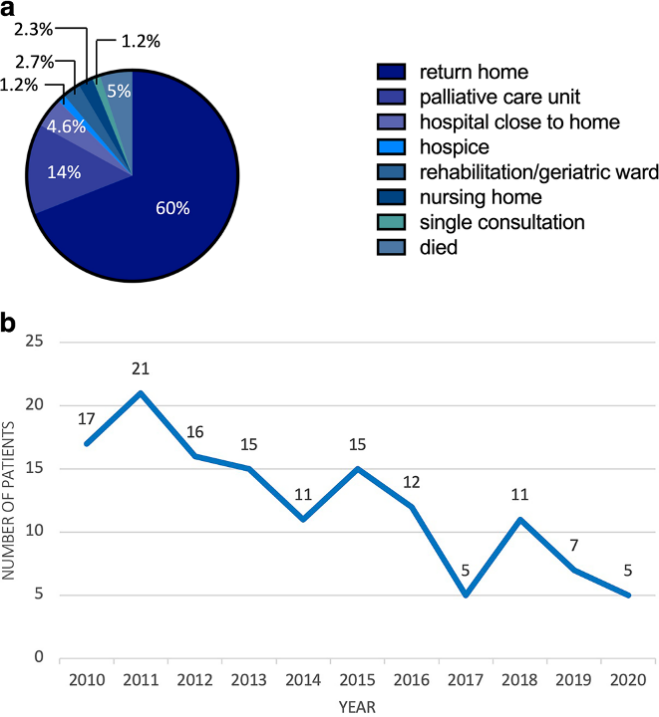
Follow-up. **a** Postdischarge care with percentage numbers of patients admitted to the respective facilities. **b** Numbers of patients dying during their stay on the radiation oncology ward. There is a significant difference between the period prior to the implementation of the palliative care consultation service ( 2010–2015) and the following time span (2016–2020; *p* = 0.009)

Since the implementation of the PCCS, the number of patients treated cooperatively increased from 97 patients in 2015 to 251 in 2021 (Fig. 2b). Most patients were able to return home with only a minority dying during their stay in the acute care hospital (Fig. 3a). Concerning our in-house PC unit, 478 patients in total have been treated since July 1, 2019, 58 of whom were referred from the RO ward. Of these 58 patients, 24 died on the PC unit, 12 were transferred to a hospice, 11 were transferred to another hospital/rehabilitation clinic, and 11 were discharged home. Over time, the number of patients dying on the RO ward decreased, with a significant difference concerning deaths between the time periods 2010–2015 (median: 15.5 deaths/year) and 2016–2020 (median: 7 deaths/year; *p* = 0.009, using the exact sampling distribution of U) indicating a strong effect (Z = −2.482; r = 0.748, Fig. 3b).

### Survey

**Fig. 4:**
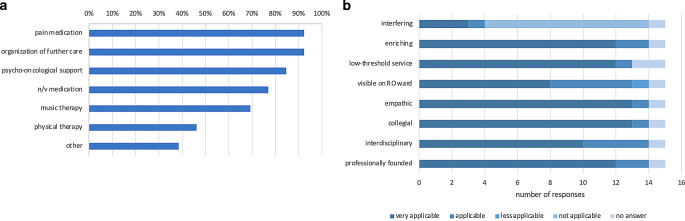
Survey. **a** Indication for palliative care consultation service (PCCS) usage and **b** perception of the PCCS among radiation oncology (RO) residents. Multiple answers were possible for indications of PCCS. *n/v* nausea/vomiting

In total, 15 participants answered the survey with 40% being female and 60% being male and a median age of 25–30 years (53.3%; 30–35 years: 26.7%; ≥ 36 years: 20%). Most residents estimated their palliative knowledge to be extensive (21.4%) or mediocre (78.6%). The PCCS was well known (100%) and used often (7.7%) or very often (84.6%) during the ward rotation. Indications for PCCS consultations were diverse (Fig. 4a) and focused on pain medication (92.3%), organization of further care (92.3%), and psycho-oncological support (84.6%). Overall, the PCCS was seen positively with a vast majority of residents agreeing on adjective like “enriching”, “empathic”, “collegial”, “professionally founded”, and a “low threshold for consultation” (Fig. 4b). However, three and one respondents found the statement to be “very applicable” or “applicable” that the PCCS is interfering with their clinical/radio-oncological routine. All participants agreed that collaboration with the PCCS resulted in a more extensive and more profound knowledge in PC (100%).

## Discussion

The hereby presented analysis demonstrates feasibility and efficacy of a structured cooperation between a radiation oncology department and a palliative care service. The main advantages lie within a supply of adequate PC for the individual patient and the possibility to provide knowledge on PC for the treating physicians.

In addition to that, a strong cooperation promotes acceptance of PC by the team, as well as by all patients on the ward [13]. PC is of key importance in a RO department. An assessment of PCCS demands in an acute care hospital demonstrated the RT department to be among the three most frequent users [12]. This is mirrored by a continuous rise in the number of radio-oncological patients visited by the PCCS in our study; starting from 97 patients in 2015 and reaching 251 in 2021. Despite that increase, the number of patients dying on the RO ward decreased concomitantly which is most likely due to the timely referral of patients to appropriate facilities other than the acute care hospital. Nevertheless, the integration of palliative services is often postponed due to multiple reasons: an analysis at the university hospital Munich revealed the majority of PCCS contacts to be initiated in the last week of the patient’s life [14]. In contrast, the current algorithm with regular ward rounds may enable a valuable cooperation before “end-of-life care” and strongly supports the concept of early integration, which has been identified as superior regarding guidance of further care, patient’s quality of life, and care costs [15–17]. Focusing on patients and their relatives, honest communication on the expected course of disease, and the limited life-expectancy may facilitate integration of PC [11].

It has to be noted that the symptom burden of palliative patients may be prone to changes during RT and demands for a dynamic care approach: An assessment of patient-reported outcomes in palliative patients revealed the most prominent (clinical relevant) symptoms before the start of RT to be an impairment of general wellbeing (62.8%), pain (62.8%), tiredness (60.0%), lack of appetite (40.0%), and anxiety (38.0%) [7]. After completion of RT, symptoms changed with a significant higher percentage of lack of appetite (60%, *p* = 0.006) in contrast to a significant decrease in pain (42.8%, *p* = 0.033) [7]. In comparison, the percentage of patients suffering from pain at the beginning of PCCS integration in our analysis was relatively low (20.4%) which may be due to the abilities of the treating radiation oncologists to manage pain medication.

PC as well as palliative RT is a pivotal part of the educational program for residents as proposed by the DEGRO [18]. This topic is not limited to radiation itself but also encompasses pain medication, supportive care, psycho-oncology, and knowledge on hospice care. Correspondingly, palliative RO is taught at 87.5% of all universities as part of the curriculum for medical students [19], which paves the way for a better understanding.

However, a North American survey reported that 79% of residents judged their training in PC to be not or only partially sufficient [20]. Concerning different topics, deficits were reported in various physiological, psychological, social, and legal domains as well as in the planning of current treatment goals and further care planning with the highest insecurity in the ability to initiate a depression treatment, rotate opioids, manage fatigue, anorexia, and insomnia [20]. Dedicated training in PC exceeding 5 h was associated with higher self-assessed competence in all domains [20]. Thus, a minimal teaching intervention may significantly enhance PCcompetences. The call for a more intensive training in PC is common in the literature: a systemic review found this statement to be present in 89.4% of publications [21].

**Fig. 5:**
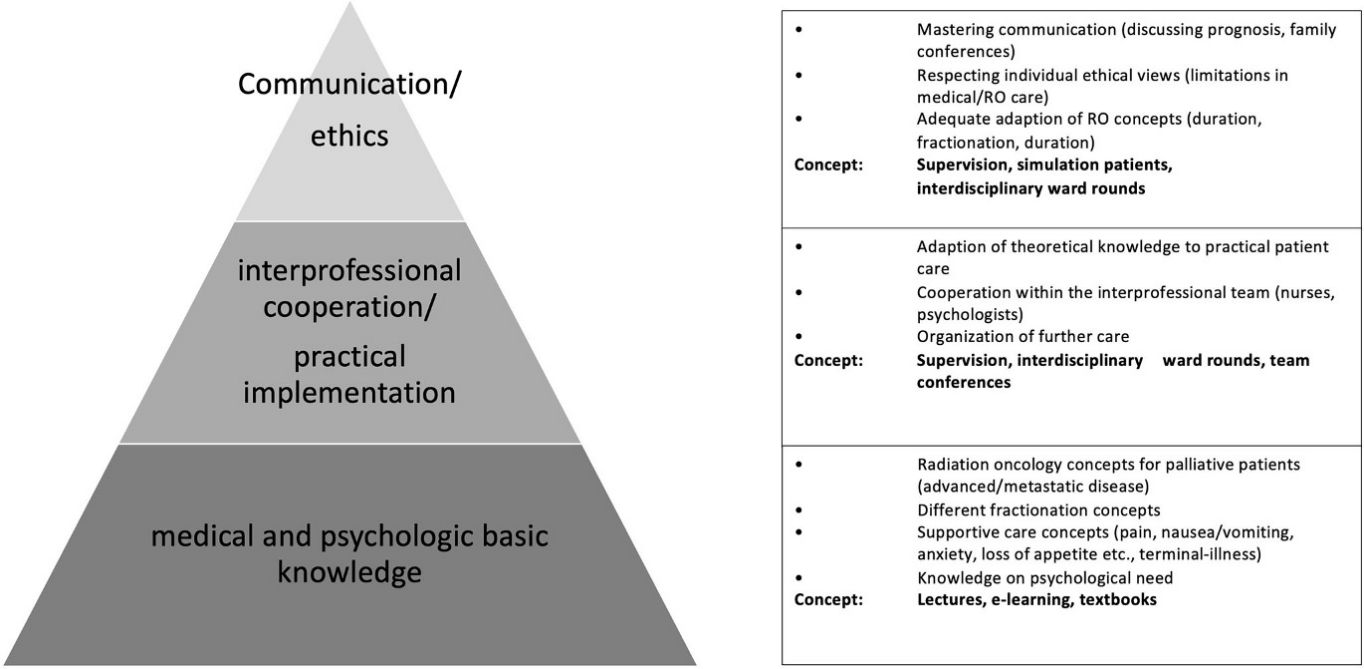
Basic curriculum for palliative care education in radiation oncology. Pyramid symbolizes consecutive learning steps which are to be mastered for adequate palliative care. *Right* *table*: Further details and suggested teaching concepts for each learning step. *RO* radiation oncology

Built on the results of the current evaluation and the presented survey, we developed a model curriculum for PC in RO (Fig. 5). It is intended as a multistep process starting from theoretical knowledge (base of the pyramid) and reaching practical use (top of the pyramid). It has to be emphasized that basic knowledge on PC should be introduced early, preferably during medical school. The hereby described protocol of regular ward rounds may assist in the advanced learning steps (top levels of the pyramid) beyond textbook experience. With its basic character, the concept may be easily adapted to multinational educational programs and addresses both residents’ need for information and supervision during daily clinical care. However, it demands profound knowledge of the supervising senior physicians, sufficient time for teaching, and effective interdisciplinary cooperation with a PCCS .

The current work has some limitations being a retrospective and monocentric analysis. In spite of that, patient numbers exceed 1000, being larger than many collectives in the literature. Unfortunately, follow-up data on mortality after discharge were incomplete which prevented a decisive survival analysis. The decision to integrate PCCS into patient care was done on an individual basis by the treating radiation oncologist, a process which may be prone to selection bias. In addition, there has been no simultaneous control group treated without the help of the PCCS, which hampers estimation of the precise impact of the described concept. This is of particular interest regarding the influence on RT treatment strategies. Being a consultation service, the PCCS did not interfere directly with decisions on RT duration and fractionation but, due to the close cooperation between the two departments, an indirect influence cannot be excluded. This question has to be addressed in future (prospective) trials.

Finally, PC in RO has to pursue multiple objectives in the years to come: (1) enlarge knowledge among treating physicians and nurses, (2) increase awareness towards individual needs of palliative patients, and (3) provide adequate guidance to PCCS on a broader scale. The presented cooperative strategy proved to be innovative and successful, but additional programs will be needed in the future.

## Supplementary Information

Supplementary Fig. 1: Development of pain. Pain intensity as given by patients on a numeric rating scale at the time of admission (mean: 5.68; median: 6; range: 2–10) and 72 h after admission (mean: 2.53; median: 2; range: 0–8).
