# Supplementary figures and images for: Palliative care on the radiation oncology ward—improvements in clinical care through interdisciplinary ward rounds
Source: Strahlenther Onkol. 2022 Aug 11;199(3):251–7. doi: 10.1007/s00066-022-01989-0 (PMC9938032; doi:10.1007/s00066-022-01989-0)

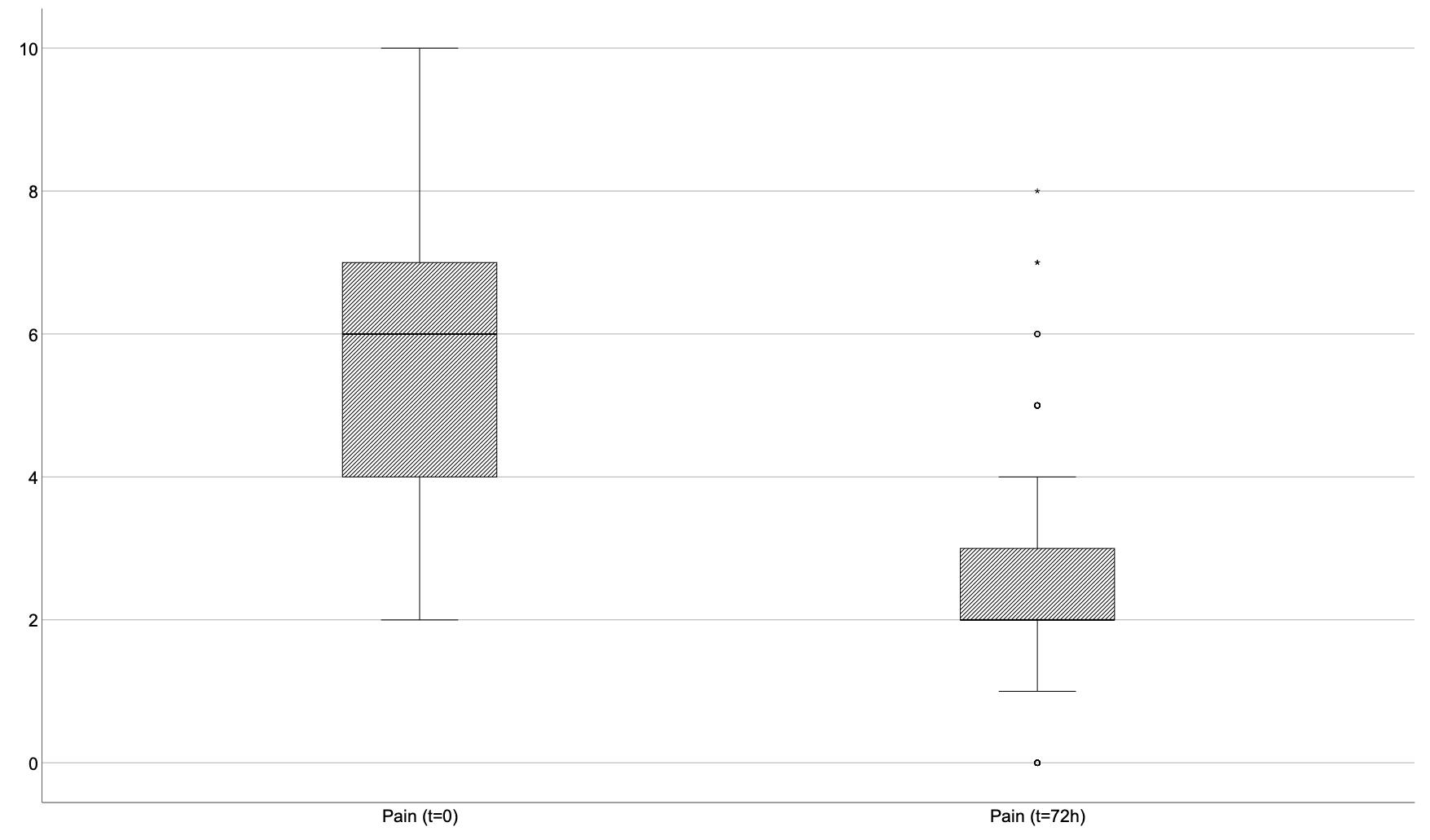

Supplement: Supplementary file 1 — Supplementary Fig. 1: Development of pain. Pain intensity as given by patients on a numeric rating scale at the time of admission (mean: 5.68; median: 6; range: 2–10) and 72 h after admission (mean: 2.53; median: 2; range: 0–8). [file 66_2022_1989_MOESM1_ESM.jpg]
